# Patient Characteristics That Influence the Experience of Patient Participation in Therapeutic Decision Making

**Published:** 2019-08

**Authors:** Yujeong KIM, Eunmi LEE, Mee Kyung LEE, Mi Young KIM

**Affiliations:** 1. College of Nursing, Kyungpook National University, 680, Gukchabosang-ro, Jung-gu, Daegu, South Korea; 2. Department of Nursing, Hoseo University, 79-20, Hoseo-ro, Baebang-eup, Asan-si, Chungcheongnam-do, South Korea; 3. School of Nursing, University of Washington, Washington, Seattle, WA, USA; 4. College of Nursing, Eulji University, Seoul, South Korea

## Dear Editor-in-Chief

Patient-centered care effectively represents the social change from a disease-centered model, where healthcare providers make every decision in the overall treatment process, to a patient-centered model, where patients participate actively and make decisions about their own therapeutic procedures (1). This change has induced patients to make therapeutic decisions that reflect their needs and preferences using their own knowledge, values, beliefs, and cultural backgrounds that are considered and respected by healthcare providers. Patient participation promotes trust and cooperation in the relationship between the healthcare providers and patients and protects patients’ rights.

Many studies have supported the utilization of patient safety tools developed based on patients’ perspectives (2) and indicated that patient participation acts as a buffer for patients’ safety (3). In Korea, patient participation and communication in the healthcare environments need to be emphasized and practiced (4). If patients participate in therapeutic decision making based on information shared between healthcare providers and patients, they can reach a mutual agreement about decisions that are made in the therapeutic procedures instead of adopting a passive attitude, and as a result, their level of satisfaction with the healthcare service will increase (5). If patient participation is practiced while no appropriate relationship is formed between patients and the healthcare providers or while no sufficient information on healthcare service is provided, it will be difficult to fulfill the fundamental objective of patient-centered treatment. In general, patients wish to know the results or available options of treatment methods and to make choices and decisions about treatment methods for themselves (6). When the disease is severe or the treatment result is highly uncertain, however, they may feel reluctant to participate in therapeutic decision-making. Rather, they may merely entrust doctors completely with the decision (7).

It is necessary, therefore, to examine the extent to which patients perceive that they participate in making therapeutic decisions.

This study used the raw data of the sixth Korea National Health and Nutrition Examination Survey (KNHANES VI) conducted in 2015. The survey sampled 6,823 adults, and 4,443 of them were included in this analysis. 11.5% of participants answered that they did not participate in therapeutic decision-making. In the optimal model, household income, occupation, severe chronic diseases, and family history of chronic diseases were excluded, and only age, education level, subjective health status, and self-care as indicated on the EQ-5D were used as response variables.

Patient participation in therapeutic decision-making increased with increase in age (β=.11, *P*<.001) and better subjective health status (β=.06, *P* <.001). On the other hand, patients participated more actively in therapeutic decision making when education level (β= −.15, *P* <.001) and self-care (β= .05, *P* =.001) score were lower. Hence, it is important to utilize information and communication methods in consideration of patient characteristics in order to improve patient participation, patient safety, and therapeutic results. It has been reported that when medical information is provided through various means, it enhances patients’ understanding and thereby induces their active participation (8). It is necessary to provide patients who are younger and of poor health status, who are highly educated, and who are capable of engaging in efficient self-care with opportunities to participate in therapeutic decision making more actively. A prospective study on intervention strategy to increase opportunities of patient participation in therapeutic decision-making is suggested.
